# Integrated analysis of bulk and single-cell RNA-seq reveals the role of MYC signaling in lung adenocarcinoma

**DOI:** 10.3389/fgene.2022.1021978

**Published:** 2022-10-10

**Authors:** Lu Hao, Qiuyan Chen, Xi Chen, Qing Zhou

**Affiliations:** ^1^ Science and Education Department, Shenzhen Baoan Shiyan People’s Hospital, Shenzhen, China; ^2^ Central Laboratory, The People’s Hospital of Baoan Shenzhen, The Second Affiliated Hospital of Shenzhen University, Shenzhen, China

**Keywords:** MYC, lung adenocarcinoma, prognosis, tumor immunity, tumor stemness, cell proliferation, cell differentiation

## Abstract

MYC is one of the well-known oncogenes, and its important role in cancer still remains largely unknown. We obtained lung adenocarcinoma (LUAD) multi-omics data including genome, transcriptome, and single-cell sequencing data from multiple cohorts. We calculated the GSVA score of the MYC target v1 using the ssGSEA method, and obtained the genes highly correlated with this score by Spearman correlation analysis. Subsequent hierarchical clustering divided these genes into two gene sets highly associated with MYC signaling (S1 and S2). Unsupervised clustering based on these genes divided the LUAD samples into two distinct subgroups, namely, the MYC signaling inhibition group (C1) and activation group (C2). The MCP counter package in R was used to assess tumor immune cell infiltration abundance and ssGSEA was used to calculate gene set scores. The scRNA-seq was used to verify the association of MYC signaling to cell differentiation. We observed significant differences in prognosis, clinical characteristics, immune microenvironment, and genomic alterations between MYC signaling inhibition and MYC signaling activation groups. MYC-signaling is associated with genomic instability and can mediate the immunosuppressive microenvironment and promote cell proliferation, tumor stemness. Moreover, MYC-signaling activation is also subject to complex post-transcriptional regulation and is highly associated with cell differentiation. In conclusion, MYC signaling is closely related to the genomic instability, genetic alteration and regulation, the immune microenvironment landscape, cell differentiation, and disease survival in LUAD. The findings of this study provide a valuable reference to revealing the mechanism of cancer-promoting action of MYC in LUAD.

## Introduction

Lung cancer is the most common malignant tumor of the respiratory system, and the basic and clinical research on lung cancer is increasingly attracting attention (Mok et al., 2019; Song et al., 2020; Song et al., 2022). Although the current research on the pathogenesis of lung cancer has made great progress, but the clinical treatment effect of lung cancer is still not satisfactory, and the long-term survival rate of lung cancer still has great room for improvement. With the deepening of research, molecular biology has been widely used in the field of lung cancer research, which not only provides many new methods for lung cancer research, but also makes the diagnosis and treatment of lung cancer into a new stage. According to the different biological characteristics, lung cancer is often divided into small cell lung cancer (SCLC) and non-small cell lung cancer (NSCLC) in clinical, among which the latter accounts for about 85% of all lung cancer patients (Cheng et al., 2019). Lung adenocarcinoma (LUAD) is the most common pathological subtype of lung cancer (Kim et al., 2019; Song et al., 2021). Lung cancer is a highly heterogeneous tumor, and lung cancer occurrence is a multi-gene, multi-factor joint regulation, multi-stage and multi-step process (Rajagopalan et al., 2018).A large number of molecular abnormalities and the mechanism of action remain to be explored.

**FIGURE 1:**
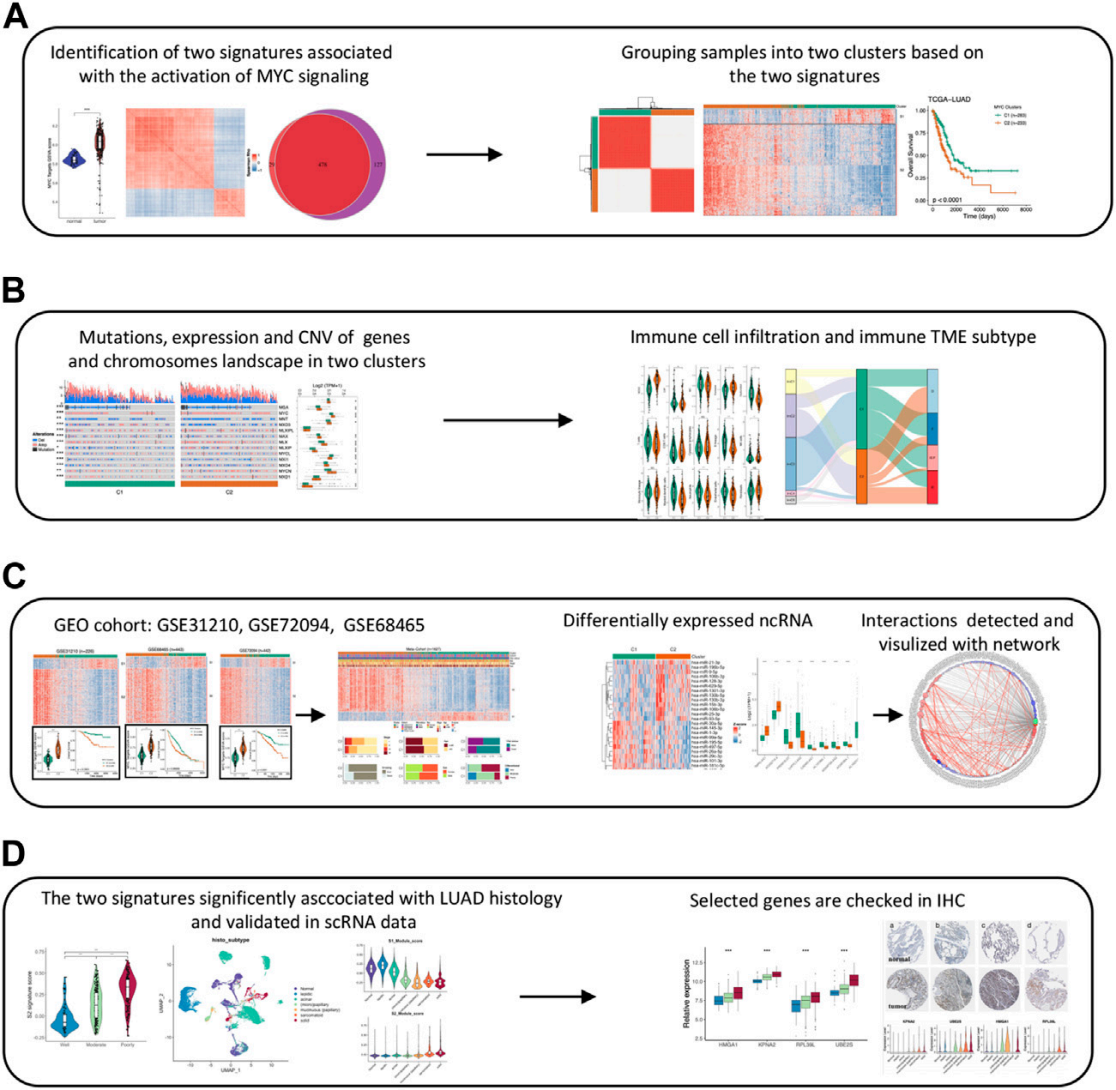
The work flow chart of this study. **(A)** Identifying MYC signaling related genes and clustering LUAD samples. **(B)** Analyzing the differences in multiple levels (Genome, transcriptome, and immune infiltration) between two clusters. **(C)** Validating the robustness of the two MYC signatures and constructing the network of differentially expressed lncRNAs and miRNAs. **(D)** Verifying signatures association with LUAD cell differention in scRNAseq data and IHC.

The MYC gene family and its products are involved in the regulation of cell growth, differentiation, and programmed death, and play important roles in the formation of various tumors (Vo et al., 2016). Previous studies have shown that MYC can affect the cell cycle progression, and its amplification and overexpression can lead to c-Myc proto-oncogene activation, which subsequently promotes tumorigenesis and progression (King et al., 2016; Lee et al., 2016).It can also regulate the expression of VEGF, to control the angiogenesis (Thompson et al., 2017). MYC, acting as a transcription factor, can regulate the expression of a large number of genes in tumors. It can act as an amplifier that globally upregulates the expression of protein-coding genes within cancer cells. So with a slight MYC expression disorder, it is possible to promote cancer cell evolution (Jing et al., 2016; Wang et al., 2019; Poh et al., 2019). Recent advances in high-throughput sequencing technologies, such as whole-genome sequencing, have allowed us to analyze tumors in unprecedented depth, especially with the single-cell sequencing (scRNA-seq) technologies that have emerged in recent years (Jiang et al., 2022; Becht et al., 2018; Zhang et al., 2021). Among them, scRNA-seq is a new technology for high-throughput sequencing of mRNA at the single-cell level, studying the overall level of gene expression for individual cells. Given the non-negligible and important role of MYC in cancer cell growth, proliferation, and differentiation, this study innovativelyused LUAD multi-omics data from multiple cohorts to systematicallyinvestigate the relevance of transcriptional profile expression, genome instability, genetic alteration and regulation, immune microenvironment landscape, cell differentiation, and disease survival in Halkmark MYC target V1 gene sets by integrating bulk and single-cell RNA sequencing data. Figure 1 showed the workflow of this study. This study indicated significant differences in prognosis, clinical characteristics, immune microenvironment, and genomic alterations between MYC signaling inhibition and MYC signaling activation groups. MYC-signaling is associated with genomic instability and can mediate the immunosuppressive microenvironment and promote cell proliferation, tumor stemness. Moreover, MYC-signaling activation is also subject to complex post-transcriptional regulation and is highly associated with cancer cell differentiation. Take together, the findings of this study provide a valuable reference to revealing the mechanism of cancer-promoting action of MYC in LUAD.

## Materials and methods

### Data sources and sample collection

Expression profile data (tpm, counts, miRNA isoform) and clinical information for TCGA-LUAD (*n* = 516) were downloaded from the GDC (https://portal.gdc.cancer.gov/). To avoid batch effects, the counts and tpm data that we used were directly derived from the STAR-counts workflow type, and were subsequently log2-transformed on the TPM data. Mutation data and copy number variation (CNV) data for the TCGA-LUAD dataset were also downloaded from the cBioPortal (www.cbioportal.org/). Three independent LUAD cohorts were collected from the GEO database (https://www.ncbi.nlm.nih.gov/geo/) asexternal validations, respectively, GSE68465 (*n* = 443) (Shedden et al., 2008), GSE72094 (n = 442) (Schabath et al., 2016), and GSE31210 (*n* = 226) (Okayama et al., 2012; Yamauchi et al., 2012). For processing the GEO data, we refer to the method of (Song et al. 2022). The LUAD GEO dataset included in this study was mainly considered based on the sample size. The above three LUAD cohorts have a substantial number of cases, which is an important basis for their inclusion in this study. The clinicopathological parameters of LUAD patients in the TCGA and GEO cohorts should be provided in the Supplementary Table S1.

### Definition of signature genes

Pathways for MSigDB database were acquired using the“msigdbr” package in R. The enrichment score for the pathway “HALLMARK_MYC_TARGETS_V1” was calculated using the ssGSEA algorithm of the “GSVA” package in R, and the genes highly correlated with this score were obtained by Spearman correlation analysis, with the threshold set as: Rho> 0.5 and adj.*p* value <1e-3. Genes were subsequently filtered using univariate cox regression analysis and log rank test with *p* value≤0.05 as the threshold, and the two lists of genes obtained were set as intersection, and shared genes were considered as survival-related genes. We divided the resulting gene set into S1 signature set and S2 signature set by hierarchical clustering. All the genes in S1 were negatively correlated with HALLMARK_MYC_TARGETS_V1 pathway score and HR < 1, while S2 was all positively correlated with HALLMARK_MYC_TARGETS_V1 pathway score and HR > 1. Therefore, S1 signature was thought to be associated with MYC signaling inhibition, and S2 signature is associated with MYC signaling activation.

### Classifying samples with consensus clustering

Samples were consistently clustered using the “ConsensusClusterPlus” package in R (Qiu et al., 2021), with the parameters set to: distance = “euclidean”, clusterAlg = “km,” maxK = 5, reps = 100, pItem = 0.8, and the remaining parameters took the default values. And sampels could be most distinctly classified when k = 2. After checking the expression level of the two signatures we previously identified in these two cluters, reasonably, we defined samples with highly expressed S1 signature genes as group C1 (MYC signaling inhibition group). Conversely, samples with highly expressed S2 signature genes were defined as group C2 (MYC signaling activation group).

### Analysis of the genomic variability

We used “data_mutations_extended.txt” downloaded from ciBioPortal to analyse the mutation landscape of two clusters. Non-silenced SNV was analyzed using the “maftools” R package. We focused onMYC gene family (MYC, MYCN, MYCL) and pathway core genes and genes listed as cancer driver genes by OncoKB(https://www.oncokb.org/cancerGenes). The Fisher test of genes mutated in at least 30 samples were also performed using the mafCompare algorithm to yield genes with significant differences in mutation frequency in the two groups. Copy number variations of related genes were analysized using “data_CNA.txt” data from ciBioPortal. Among them, the CNV state of genes is divided into −2, −1, 0, 1, 2, and 0 represents no CNV, 1 and 2 represent copy number amplification, and -1 and −2 represent copy number loss. Copy number variation at chromosome level were directly extracted from the data_clinical_sample.txt, and only the top 10 most significantly variated chromosome arms between C1 and C2 clusters were visualized. All the statistics of genomic variability were performed with two-sided Fisher’s exact test.

### Description of the tumor microenvironment

The scores of 10 typical immune cells, including T cells, CD8 T cells, CTL, B cells, NK, and monocytes, were calculated using MCP-counter. From a previous study (Bagaev et al., 2021), data including purity, intratumor heterogeneity, aneuploidy score, homologous recombination defects, BCR.Shannon, TCR.Shannon, M1/M2 macrophage were obtained. To further evaluate the impact of MYC on the immune microenvironment, we used TIDE (http://tide.dfci.harvard.edu/) to calculate the scores of TIL for MDSC, CAF, and M2, as well as two indicators related to immunotherapy response: T-cell dysfunction and exclusion (Jiang et al., 2018).

### Differential expression analysis of genes (including mRNA, lncRNA, miRNA) and the construction of CeRNA network

Using the “DESeq2” R package, the differential expression analysis was performed (Zhao et al., 2021). The threshold was set to adj.*p* value <0.001 and |log2FoldChange|> 0.5. And the resulting log2FC and adj.*p* value were used as the colors and sizes of the nodes in the subsequent network graph drawing, respectively. Circular nodes represents lncRNA, and square nodes represents miRNA. The selected lncRNA-miRNA interaction, MYC/MYCN and-ncRNA interaction, and miRNA-MYC/MYCN interaction were predicted using the online tool RNAInter (http://www.rnainter.org/) and mirWalk (http://mirwalk.umm.uni-heidelberg.de/). For the prediction results, the drawing was performed using the “igraph” R package (Mora and Donaldson, 2011). Gray lines represent all possible interactions between ncRNA and MYC/MYCN, and red lines indicate possible interactions between ncRNA.

### Analysis of the scRNA-seq data

The expression matrix of scRNA-seq and the clinical information (such as histological type) of the samples were downloaded from the website (https://doi.org/10.24433/CO.0121060.v1) (Kumar and Song, 2022). The data contained a total of 114,489 cells from 10 LUAD samples and 10 normal lung tissue samples, and used 10x genomics for sequencing. Genes below expression in 100 cells were filtered out using the “Seurat” R package (Kumar and Song, 2022). Low-quality cells were filtered out by the criteria where the number of expressed genes was greater than 100 and less than 6,000 and the proportion of mitochondrial gene expression was less than 20. After defining and isolating epithelial cells from single cell expression profiling data of total cells, again, samples with less than 100 epithelial cells were filtered out for subsequent analysis. The top 15 principal components were used after PCA dimension reduction. Eventually we obtained 3,684 normal epithelial cells from the normal samples, and 15,477 malignant epithelial cells from the tumor samples. The signature module score was calculated using the AddModuleScore function.

### The interaction network of the Signature gene and immunohistochemical

Genes with significant differential expression between the two groups (C1 and C2 groups) and the genes belonging to S1/S2 signature set were included in the potential nodes. Associations between nodes were obtained by correlation analysis, and edges with lower associations were filtered out. The visualization was then performed using the “igraph” R package.Circle size represents the -log10 (*p*-value), and the circle color indicates the log2FoldChange for the difference analysis after C1/C2 grouping. Pictures of IHC staining derived from normal samples and LUAD samples were selected on the HPA website (https://www.proteinatlas.org/) to verify the relationship of key genes to cell differentiation. Here, “HPAanalyze” R package was used to download the high definition IHC pictures (Tran et al., 2019).

### Statistical analysis

All statistical analysis was done using R. Where the KM survival analysis was performed by log rank test using the “survival” and “survminer” R packages, and the univariate and multivariate cox were done using the basis function coxph. We filtered out samples with less than 30 days of follow-up date before performing a survival analysis. Student’s *t*-Test was used to compare the differences in gene expression levels between clusters. A *p*-value of less than 0.05 was considered statistically significant. Heatmaps were all plotted using the “ComplexHeatmap” R package.

## Results

### Identification of the two MYC signaling-associated signatures

**FIGURE 2:**
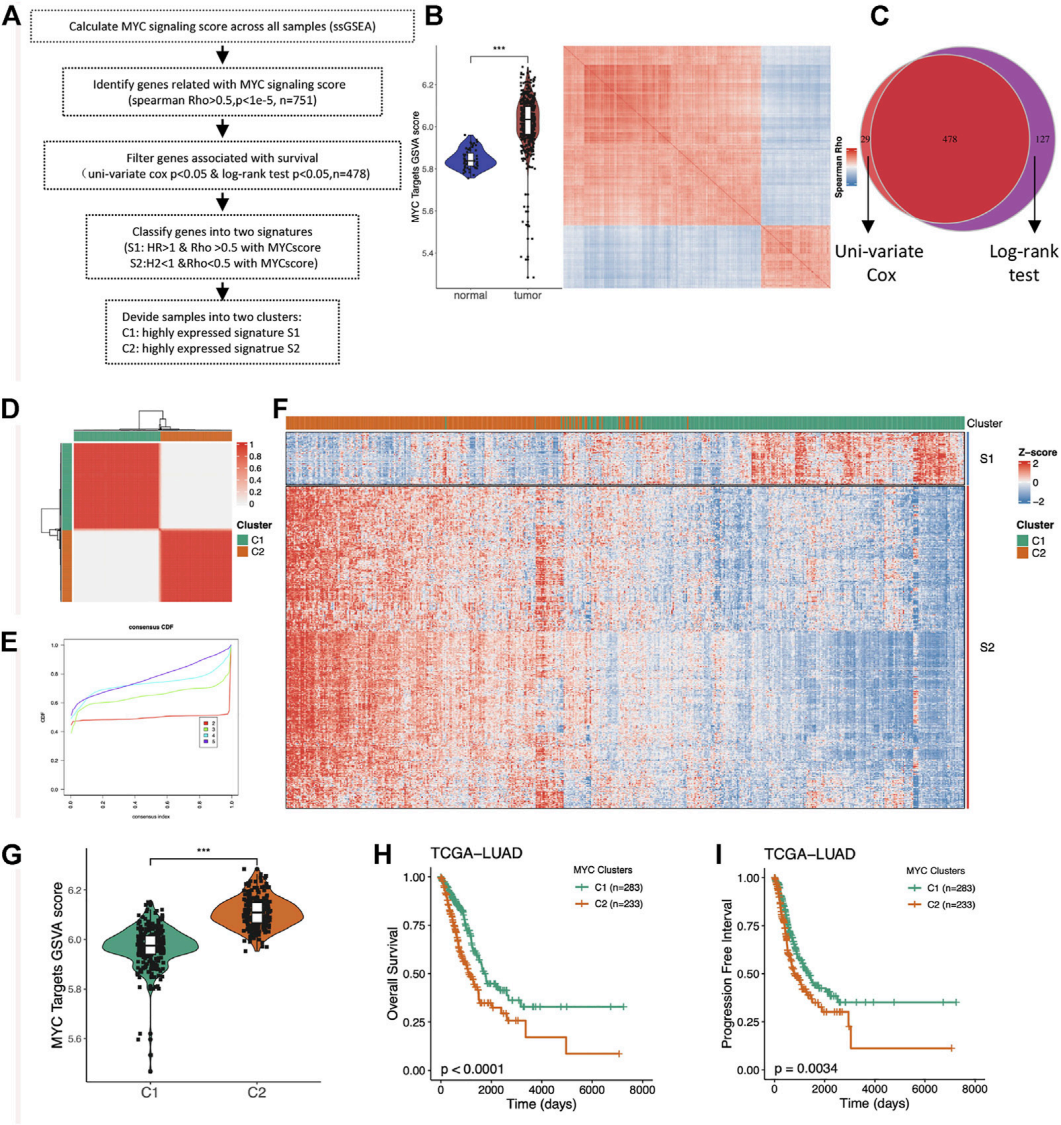
Identification of the two MYC signaling-associated signatures and unsupervised consistent clustering. **(A)** Flow chart of the sample classification. **(B)** Comparison of MYC Target GSVA scores between normal and tumor tissues in LUAD (right); Heatmap showing the hierarchical clustering of MYC Target GSVA scores-associated genes (left). **(C)** Venny plot showing survival-associated genes obtained by univariate cox analysis and log-rank test. **(D)** Unsupervised clustering divided the LUAD samples into two distinct subgroups (k = 2). **(E)** CDF and consensus index. **(F)** Heatmap showing the expression distribution of the Signature genes between the two clusters. **(G)** Comparison of MYC Target GSVA scores between the two distinct subgroups (C1 and C2). **(H)** Comparison of overall survival (OS) between C1 and C2. **(I)** Comparison of progress-free interval (PFI) between C1 and C2.

The level of MYC signaling activation cannot be simply judged by MYC gene expression and copy number amplification. Considering that the genes regulated by the same pathway are similar in their expression patterns, we assessed the degree of MYC signaling activation by looking at the overall expression levels of the MYC target genes, and obtained all the highly correlated genes by similarity analysis. We performed the subsequent analysis as to Figure 2A. We first evaluated the enrichment score of the MYC_TARGET_V1 pathway by ssGSEA algorithm, then found the genes highly related with the score through correlation analysis, and performed hierarchical clustering (Figure 2B). As we expected, these genes could be divided into two groups that were highly concordant, with one group being highly positively correlated with MYC_TARGET_V1 and the other group being highly negatively correlated. To further screen for key genes, we further filtered out 478 survival-related genes by intersection using univariate cox analysis and log rank test of the obtained genes (Figure 2C; Supplementary Table S2). Importantly, both cluster genes negatively associated with MYC_TARGET_V1 were associated with better prognosis, and both genes positively associated with MYC_TARGET_V1 were associated with worse prognosis. Therefore, we defined these two cluster gene sets as S1 and S2, respectively.

### The MYC signaling-associated signature could divide LUAD patients into two clinical clusters

Considering that the S1 and S2 genes have significantly different characteristics, we subsequently performed unsupervised consistent clustering of LUAD samples based on the expression of the signature genes, and finally obtained two clusters of samples (Figures 2D,E). One group of samples highly expressed the S1 signature gene, while the other group also highly expressed the S2 signature gene (Figure 2F), so we named it as the corresponding two C1 and C2 groups. Group C2 was the MYC signaling activation group, and group C1 was the MYC signaling inhibition group. The MYC scores were significantly different between the two groups (Figure 2G). In addition, we also found significant differences in OS (Figure 2H) and PFI (Figure 2I). This suggests important roles of MYC signaling in LUAD.

### Association of MYC signaling with Hallmark pathways and genomic variations

**FIGURE 3:**
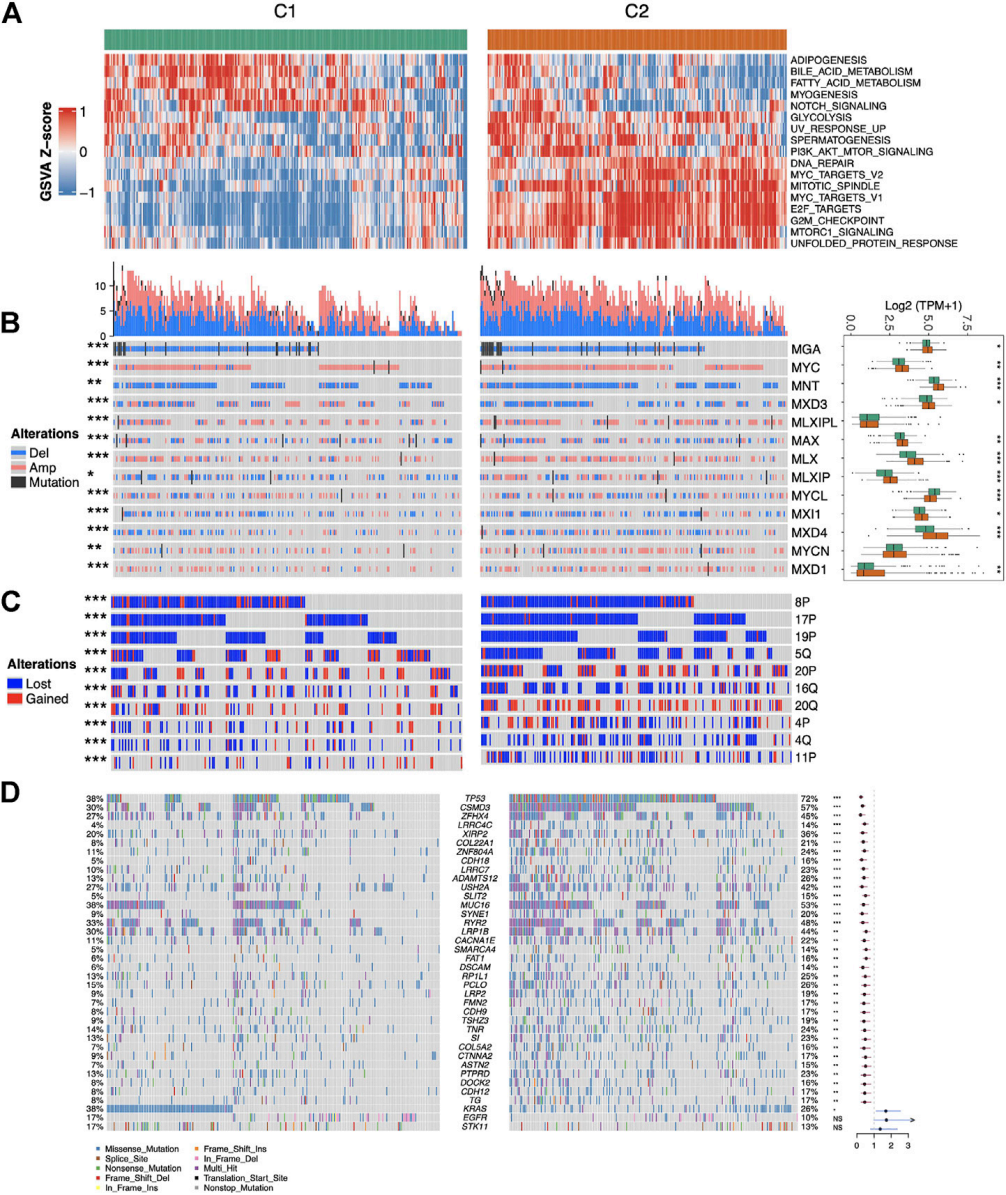
Association of MYC signaling with Hallmark pathways and genomic variations. **(A)** Heatmap of pathway scores with significant differences (*p* < 0.001) between C1 and C2. **(B)** Mutations, copy number variations and expression of core members of the MYC pathway between C1 and C2 samples. **(C)** Variation at top10 chromosome arm levels with significant differences between C1 and C2. **(D)** Waterfall plot showing the distribution of mutation characteristics of commonly mutated genes in the C1 and C2, and differently mutated genes between two groups by fisher test.

The association of MYC signaling with oncogenic pathways and genomic variants remains unclear, therefore, we investigated the GSVA score differences in Hallmark pathways between MYC signaling activation (C2) and inhibition (C1) groups. As shown in Figure 3A, in addition to the MYC and cell-cycle-related pathways, the pathways such as glycolysis and PI3K were also up-regulated in C2. Copy number variation (CNV) in all MYC pathway core genes were significantly different between C1 and C2 (fisher exact test *p* < 0.05). Specifically, these genes developed CNV more frequently in C2, and MXD3 was both primarily lost in C2 and mostly amplified in C1. MLXIP was the opposite. This suggested that CNV changes were important causes of MYC pathway activation. Meanwhile, besides MLXIP and MYCN, other genes also differed in their expression between C1 and C2. Interestingly, although MXD3 experienced more copy number loss in C2, its expression remained higher in C2 (Figure 3B). We also examined CNV differences in chromosome levels between C1 and C2 (Figure 3C). Not surprisingly, multiple chromosomes-level CNV differences exist between C1 and C2. In addition to occurring more frequently in C2, the types of variants occurring also varied, such as 5q being more amplified in C1. In LUAD, mutations in many key genes play a crucial role in tumor development. They are known as the driver genes. We examined the mutation situation between C1 and C2. The results showed that besides KRAS, EGFR, STK11 (these genes were thought to be mutually exclusive to MYC pathway activation in previous studies (Zhang et al., 2016; Mollaoglu et al., 2017)), most genes were more mutated and higher in C2 (Figure 3D). Overall, the results of this study indicate that MYC signaling is closely related with oncogenic pathways and genomic variants.

### MYC-signaling associates with genomic instability, mediates the immunosuppressive microenvironment, and promotes cell proliferation, and tumor stemness

**FIGURE 4:**
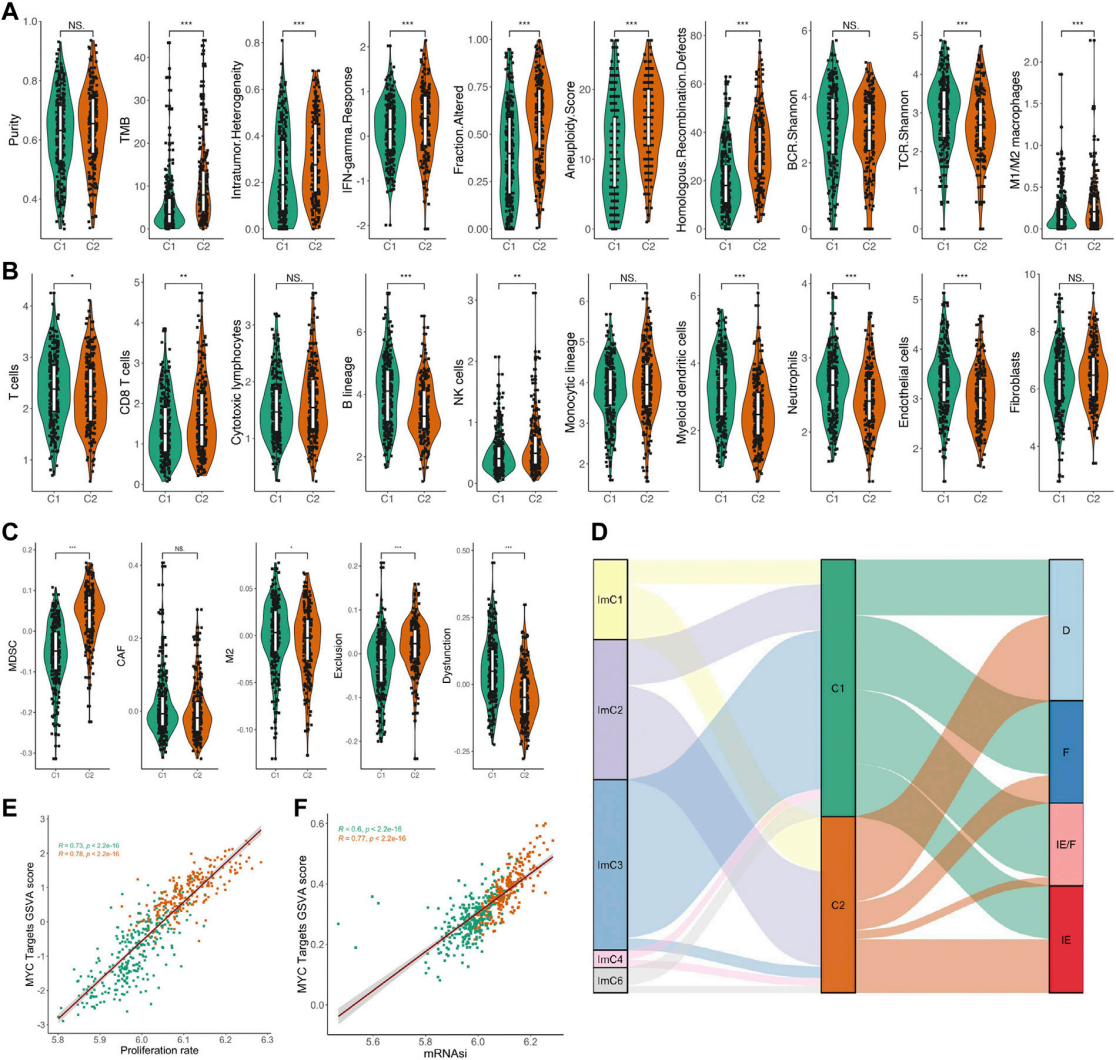
Relationship between MYC signaling and genomic instability score, immune microenvironment, cell proliferation, and tumor stemness. **(A)** Comparison of the purity, TMB, intratumor heterogeneity, IFN-gamma response, fraction altered, aneuploidy score, homologous recombination defects, BCR.Shannon, TCR.Shannon, M1/M2 macrophage between C1 and C2. **(B)** Comparison of the abundance of immune cell infiltration between C1 and C2. **(C)** Comparison of the scores of TIL for MDSC, CAF, and M2, as well as two indicators related to immunotherapy response: T-cell dysfunction and exclusion between C1 and C2. **(D)** Association of C1/C2 with two immune microenvironment types. **(E)** Correlation of the MYC Target GSVA score and cell proliferation. **(F)** Correlation of the MYC Target GSVA score and tumor stemness.

In the above analysis, we found that the MYC signaling activation group was significantly different from the inhibition group in terms of genetic mutations. From this, we further investigated the differences in genomic instability scores between the two groups. We curated a list of genomic instability scores from a previous study (Bagaev et al., 2021), which was composed of the mutation burden score, the aneuploidy score, and the HRD score. The mutation burden score was non-silent mutations per Mb. The aneuploidy score reported the total number of arm-level amplifications and deletions and was computed using ABSOLUTE. Our results indicate that the MYC signaling activation group presents a higher genomic instability score than the MYC signaling inhibition group (Figure 4A). In addition, we also found that the MYC signaling activation group also showed higher intratumoral heterogeneity, IFN-gamma response and M1/M2 macrophages and lower TCR shannon, while the tumor purity and BCR shannon did not be significantly different between the two groups (Figure 4A). To analyze the effect of MYC signaling on the immune cells and the tumor microenvironment, we calculated the infiltration levels of the 10 immune cells using the MCP counter R package and performed a statistical test with a *t*-test (Figure 4B). We found that the cells mediating tumor killing (CD8^+^ T cells, NK cells) had a higher infiltration abundance in the MYC signaling activation group. As important as the infiltration abundance of the immune cells in mediating the tumor immune response is the functional status of the immune cells, so we also evaluated indicators that reflect the immune function of LUAD with the online web tool TIDE (http://tide.dfci.harvard.edu/). The results showed that the tumor immune dysfunction score was significantly lower in the MYC signaling activation group when compared to the MYC signaling inhibition group (Figure 4C). This further suggests the importance of MYC signaling in mediating the tumor immunosuppressive microenvironment. For the tumor immune microenvironment, David Sacks et al. classified cancer samples into immune subtype in C1-C6 (Sacks et al., 2018). Similarly, Alexander Bagaev et al. defined the pan cancer sample of TCGA as four isoforms: IE, IE/F, D, and F (Bagaev et al., 2021). We explored the association between both C1/C2 groups and the tumor microenvironment of these two different differentiation methods. Coincidentally, our data suggest that ImC3 has a largely overlapping relationship with C1 (Figure 4D). This further highlights the association of MYC signaling with the tumor immunosuppressive microenvironment. Incidentally, we also explored the relationship between MYC signaling and cell proliferation and tumor stemness. Surprisingly, the MYC score had a significant correlation with both (Figures 4E,F).

### Independent validation of MYC-signaling grouping and prognosis

**FIGURE 5:**
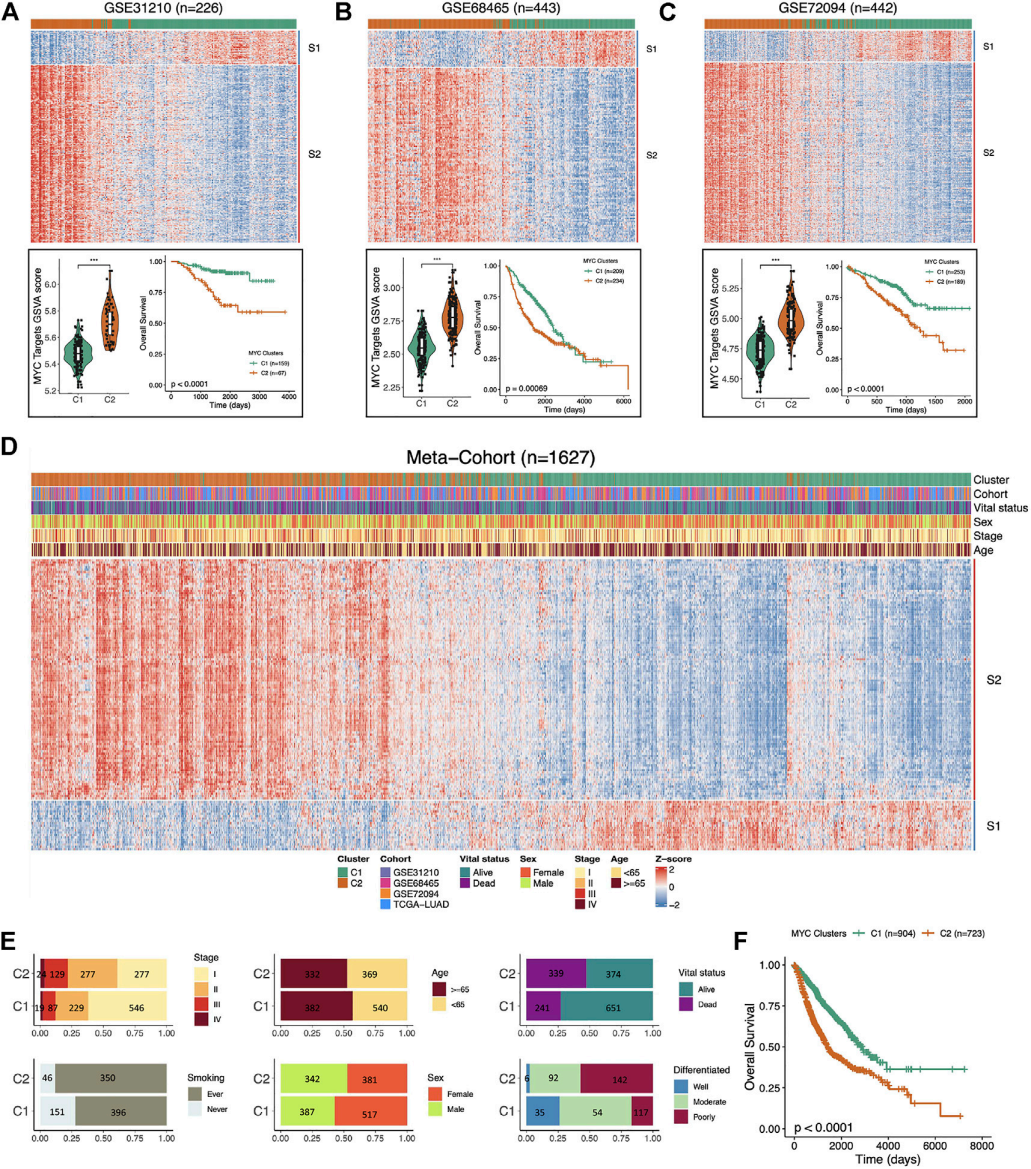
Independent validation of MYC-signaling grouping and prognosis. **(A)** The upper part the heatmap showing the expression distribution of the Signature genes between the two clusters in GSE31210. The Lower part: comparison of MYC Target GSVA scores between the two distinct subgroups (C1 and C2) in GSE31210 (right); Comparison of overall survival (OS) between C1 and C2 in GSE31210 (left). **(B)** The upper part: the heatmap showing the expression distribution of the Signature genes between the two clusters in GSE68465. The Lower part: comparison of MYC Target GSVA scores between the two distinct subgroups (C1 and C2) in GSE68465 (right); Comparison of overall survival (OS) between C1 and C2 in GSE68465 (left). **(C)** The upper part: the heatmap showing the expression distribution of the Signature genes between the two clusters in GSE72094. The Lower part: comparison of MYC Target GSVA scores between the two distinct subgroups (C1 and C2) in GSE72094 (right); Comparison of overall survival (OS) between C1 and C2 in GSE72094 (left). **(D)** The expression trend of signature genes and the distribution of the clinical characteristics of LUAD patients in the C1 and C2 in the meta-cohort (n = 1,627). **(E)** The distribution of the clinical characteristics (stage, smoking, age, sex, vital status, and grade) of LUAD patients in the C1 and C2. **(F)** Comparison of overall survival (OS) between C1 and C2 in the meta-cohort.

To verify that the two signature (S1 and S2) we defined were stable on dividing LUAD samples into C1 and C2 groups according to MYC-signaling activation levels, we used three independent GEO datasets and a meta-cohort including 1,627 caces. The results showed that the MYC-signaling grouping was robust, which could efficiently classify samples into MYC-signaling activation group (C2) and MYC-signaling inhibition (C1), and were always highly correlated with patient prognosis (Figures 5A–D). Subsequently, we also investigated the distribution of clinical characteristics between the two groups, and we found that the MYC signaling activation group had more dead patients, who had later staging and poor cell differentiation, as shown in Figure 5E. Furthermore, Figure 5F also further confirmed that MYC C2 patients had a shorter OS.

### MYC-signaling activation was subject to complex post-transcriptional regulation

**FIGURE 6:**
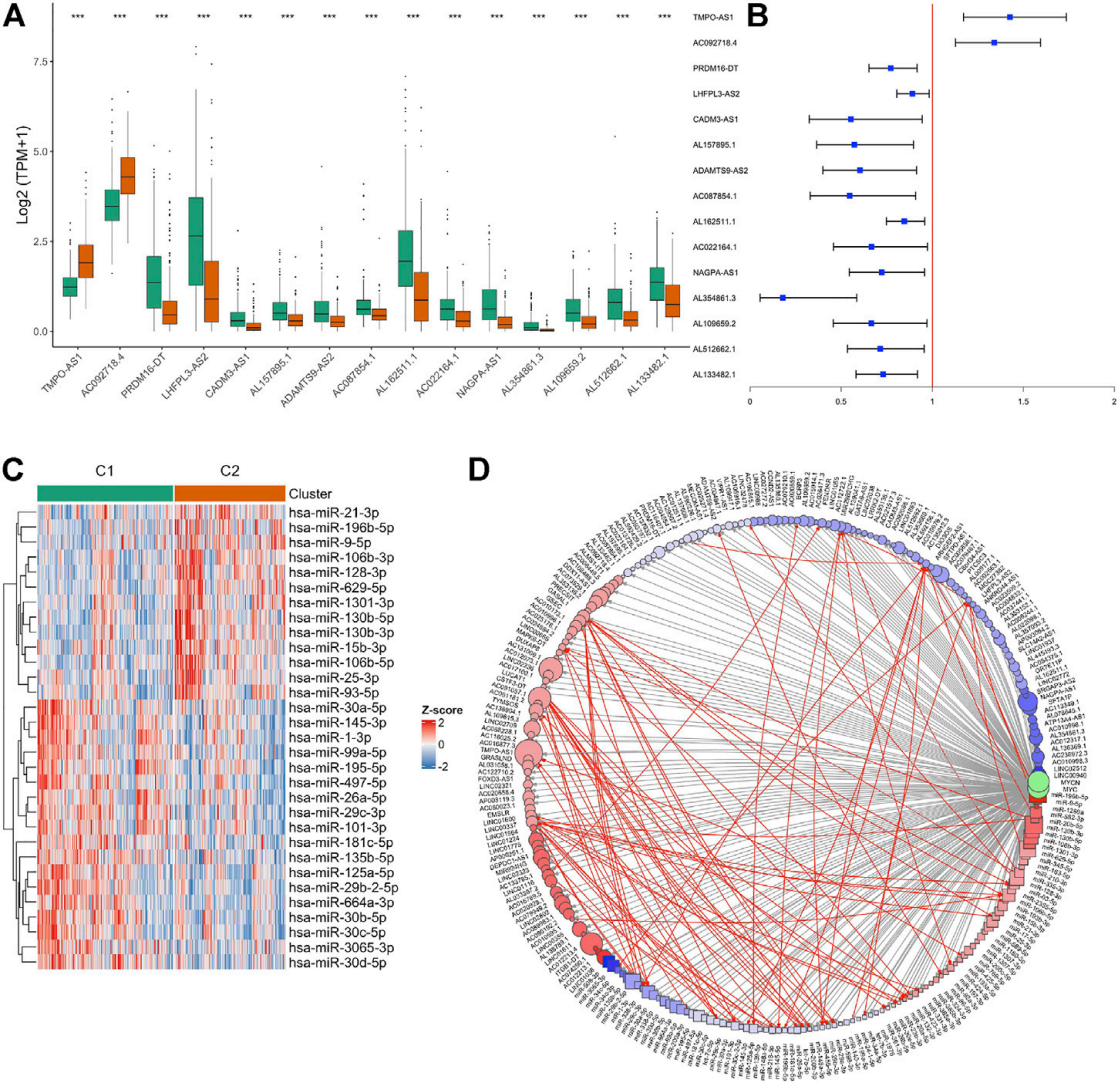
Identification of non-coding RNA associated with MYC signaling. **(A)** lncRNAs differentially expressed between the C1 and C2 groups. **(B)** Univariate Cox analysis revealed the relationship between these lncRNAs and prognosis. **(C)** miRNAs differentially expressed between the C1 and C2 groups. **(D)** Construction of CeRNA networks associated to MYC signaling. The resulting log2FC and adj.*p* value were used as the colors and sizes of the nodes in the subsequent network graph drawing, respectively. Circle represents lncRNA, and square represents miRNA.Gray lines represent all possible interactions between ncRNA and MYC/MYCN, and red lines indicate possible interactions between ncRNA.

In both the TCGA and GSE31210 data, some samples were still classified into the MYC-signaling inhibition group (C1) even with MYC experiencing copy number amplification. This suggested that MYC-signaling activation was complex regulated. Coincidentally, we found 15 lncRNAs in these two signature gene sets (S1 and S2), of which 13 belong to S1 and 2 belong to S2 (Figure 6A). And the univariate cox analysis suggested that they were all associated with prognosis (Figure 6B). In addition to lncRNA, miRNA may also play an important role in regulating MYC-signaling activation. Therefore, we also explored the differentially expressed miRNAs between the two groups (Figure 6C), and found 31 miRNAs were differentially expressed between groups. lncRNA and miRNA, mRNA may regulate gene expression through the CeRNA mechanism, and may also independently affect protein expression through other mechanisms such as acetylation. So we constructed a potential MYC/MYCN expression regulatory network (Figure 6D).

### MYC-signaling was highly correlated with cell differentiation

**FIGURE 7:**
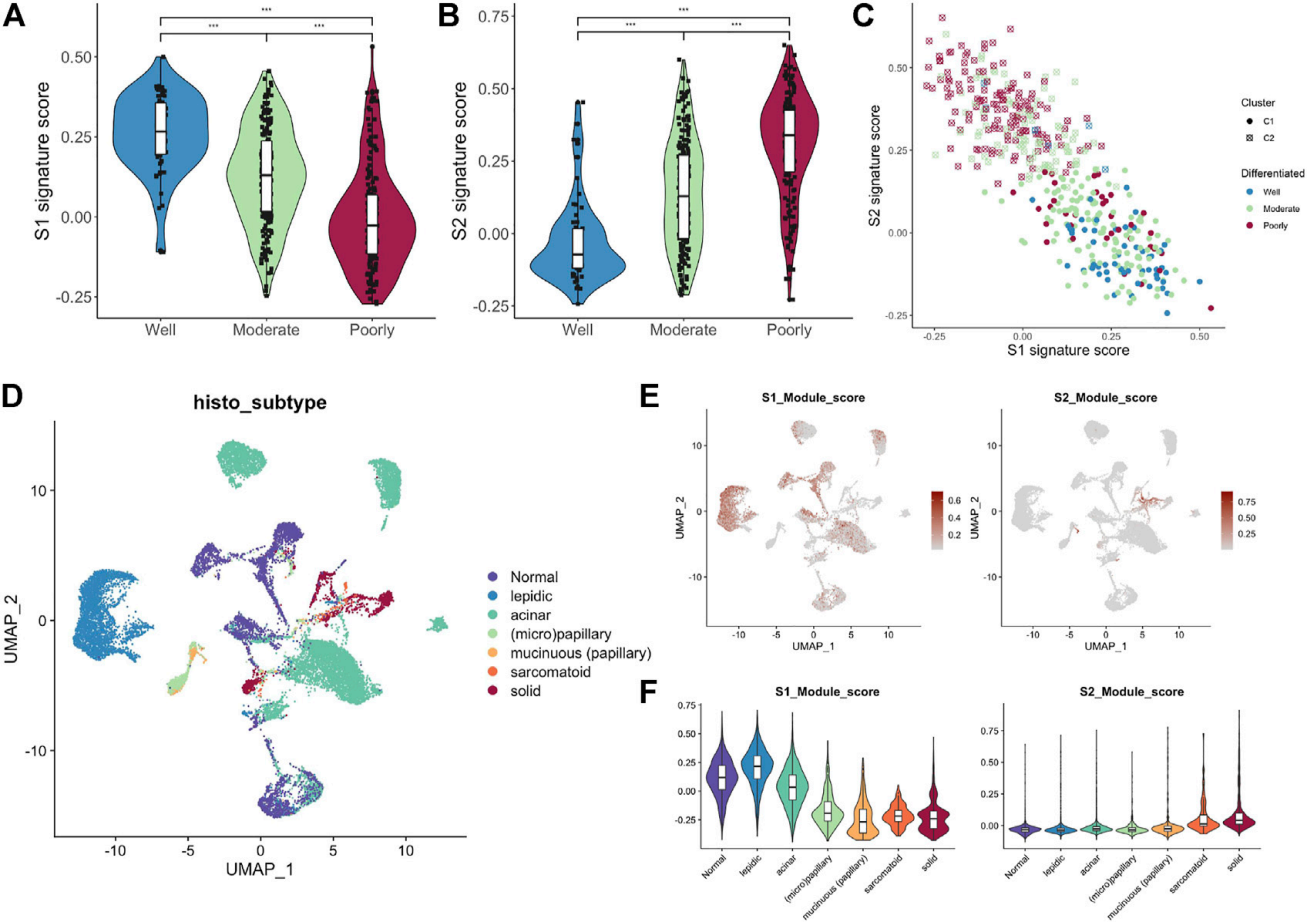
MYC-signaling is highly correlated with cell differentiation. **(A)** The relation between the S1 signature score and cell differentiation in GSE68465. **(B)** The relation between the S2 signature score and cell differentiation in GSE68465. **(C)** The connection between S1 signature score, S2 signature score, MYC signaling, and cell differentiation in GSE68465. **(D)** UMAP analysis identifies cell populations of different tissue subtypes **(E,F)** The average expression level of S1/S2 signature genes in each single cell was calculated by the AddMouduleScore algorithm.

**FIGURE 8:**
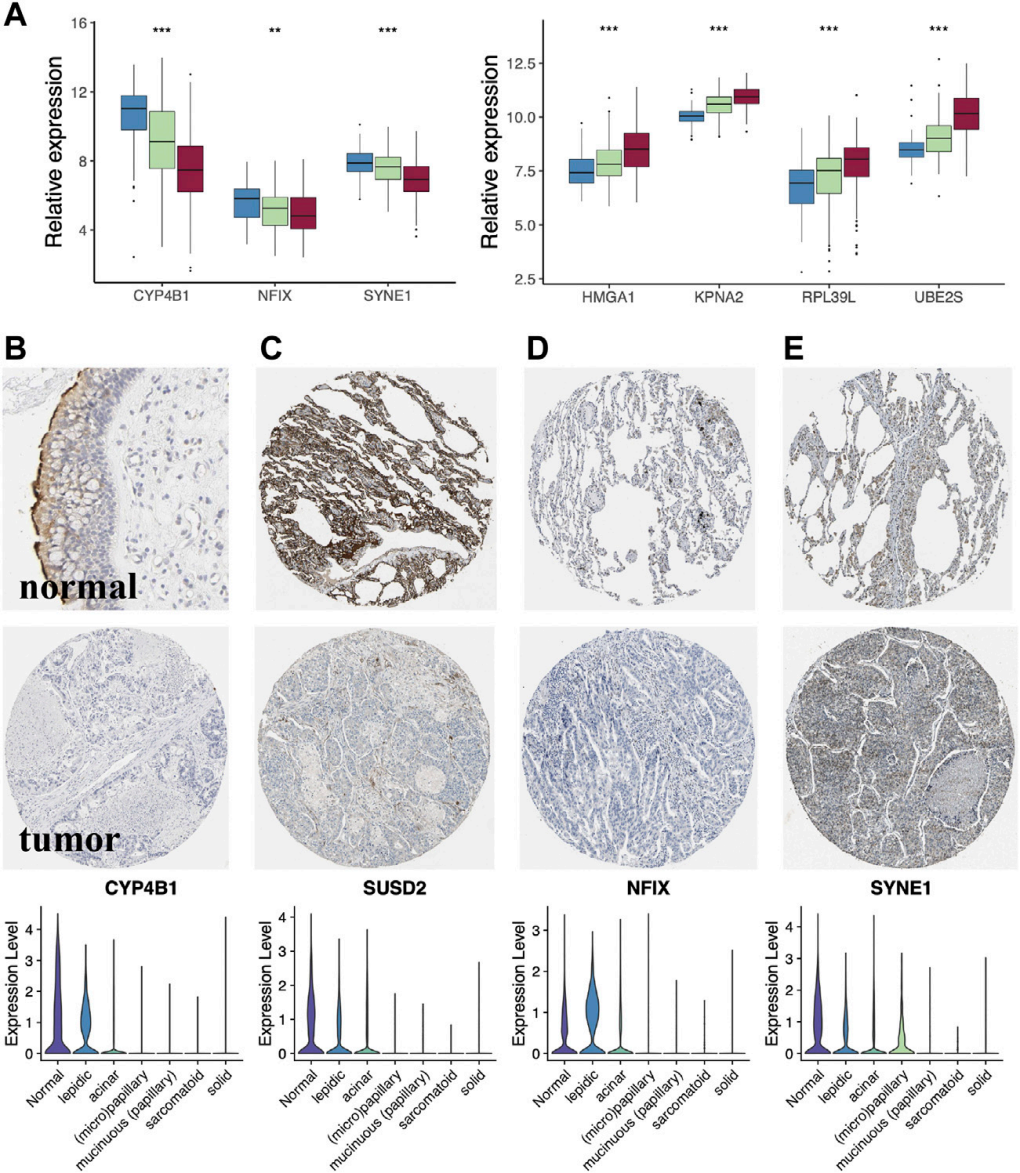
The key genes of MYC-signaling signature. **(A)** Genes highly associated with tumor cell differentiation. **(B–E)** Expression level of the key genes (CYP4B1, SUSD2, NFIX and SYNE1) in LUAD tumor tissues, normal tissues as well as in single cells with different degrees of differentiation.

**FIGURE 9:**
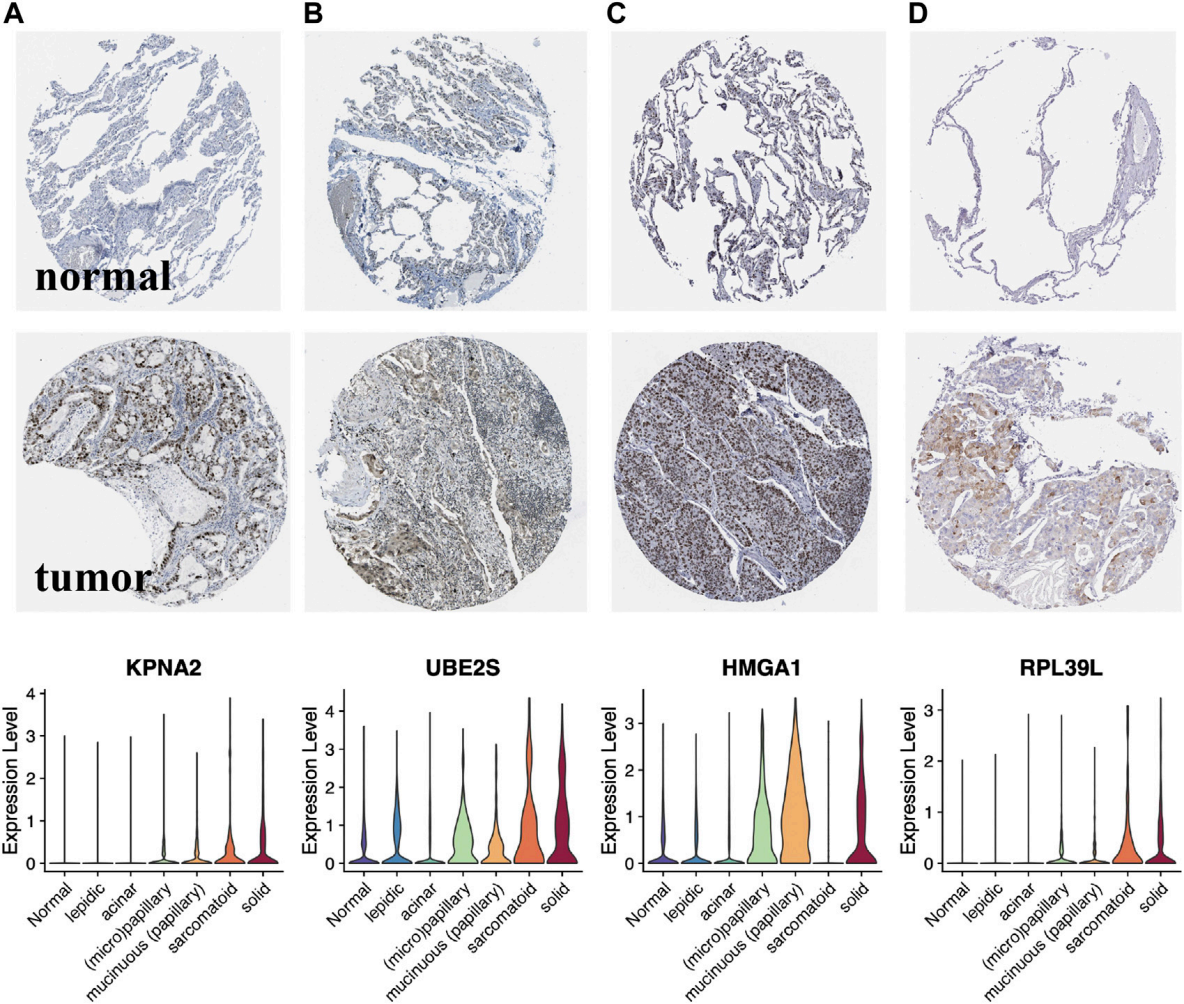
Lower expression genes in the normalrelative to the LUAD samples of MYC-signaling signature. **(A–D)** Expression level of the key genes (KPNA2, UBE2S, HMGA1 and RPL39L) in LUAD tumor tissues, normal tissues as well as in single cells with different degrees of differentiation.

Histologically, samples with highly differentiated tumor cells were highly concentrated in C1, while those with poorly differentiated cells were highly concentrated in C2. Moreover, the S1/S2 signature score can independently distinguish the tumor cell differentiation level (Figures 7A–C). To further test the significance of these two signature, epithelial cells from LUAD samples and normal lung tissue at different differentiation levels were isolated and analyzed separately. After dimensionality reduction by PCA and UMAP, we obtained 3,684 normal epithelial cells, and 15,477 malignant epithelial cells (Figure 7D). The average expression level of S1/S2 signature in each single cell was calculated by the AddMouduleScore algorithm (Figures 7E,F). The results were highly consistent with the previous findings. We found eight genes that were highly associated with cell differentiation were significantly differentially expressed in samples with different levels of differentiation in GSE68465 and showed consistent changes with the degree of differentiation (Figure 8A). Among them, CYP4B1, SUSD2, NFIX, and SYNE1 were highly expressed in normal lung epithelial cells and highly differentiated epithelial cells (Figures 8B–E bottom). The IHC staining also indicated that they had a higher expression in the normal (Figures 8B–E top) relative to the LUAD samples (Figures 8B–E middle). KPNA2, UBE2S, HMGA1, and RPL39L were highly expressed in poorly differentiated lung epithelial cells (Figures 9A–D bottom). The IHC staining also indicated that they had a lower expression in the normal (Figures 9A–D top) relative to the LUAD samples (Figures 9A–D middle). These results indicated that MYC-signaling was highly correlated with cancer cell differentiation.

## Discussion

In this study, we examined the tumor MYC_TARGET_V1 score in multiple large LUAD cohorts, and its correlation with transcriptional profile expression, genomic instability, genetic alteration and regulation, immune microenvironment landscape, cell differentiation, and disease survival. MYC, acting as a transcription factor, and a slight disturbance of MYC expression may promote cancer cell evolution. To investigate the level of MYC signaling activation, we analyzed the expression levels of the MYC gene family and pathway core genes. We found that these genes were mostly significantly different between the two groups. Further investigating the copy number variation of the core MYC pathway genes between the two groups, we found that they did not show significant differences in the copy number variation. This implies that the activation of MYC signaling is epigenetically regulated, for example, DNA methylation (Panopoulos et al., 2017).It has been shown that the turnover of Myc proteins is determined by a cascade of phosphorylation and ubiquitination events (Liu et al., 2019; Parang et al., 2017).Notably, there is still a lack of evidence on whether MYC is regulated by ncRNA. In contrast, MYC, as a transcription factor, can regulate the activation and expression of ncRNA, for example, the miR-15 and let-7 (Adams and Eischen, 2016). In the study from Hou et al.(Zhang et al., 2022), they found that the MYC/MAX-trans-activated LINC00958 could promote the malignant behavior of LUAD by recruiting HOXA1 and inducing oncogenic reprogramming.To further clarify the pathways in which MYC is involved, we calculated the enrichment scores of the 50 Hallmark pathways in MsigDB by the ssGSEA algorithm, and found differences in multiple Hallmark pathway enrichment scores between the two groups, a finding that was also consistent with previous studies. c-Myc, an important member of the MYC gene family, acts as a proto-oncogene localized to chromosome 8q24.1 and can be activated by chromosomal amplification, translocation, and rearrangement (Xu-Monette et al., 2016). In the above analysis, we also found that the MYC signaling activation group was significantly different from the inhibition group in terms of gene mutations. We further investigated the relationship between MYC signaling and the genome instability. We found that the MYC signaling activation group presented higher genomic instability scores. This result was not surprising, as reported in previous studies (Hao et al., 2016; H. Song et al., 2020).

More and more researchers have noticed the close link between tumor immune microenvironment and cancer occurrence and progression (Wang et al., 2016; Yuan et al., 2018; Tekpli et al., 2019). The MYC gene was also reported to be involved in the immune regulation of multiple tumors (Han et al., 2019; Swaminathan et al., 2020). In this study, we found that the cells mediating the tumor-killing effect had a higher infiltration abundance in the MYC signaling activation group. Further investigating the functional status of the immune cells, we found that the tumor immune dysfunction score was significantly lower in the MYC signaling activation group as compared to the MYC signaling inhibition group. This further suggests the importance of MYC signaling in mediating the tumor immunosuppressive microenvironment.Moreover, the association of MYC with immune checkpoints is also slowly being revealed. For example, Thongsuksai et al. (Sunpaweravong et al., 2022)found that NSCLC tissues significantly express more c-Myc and PD-L1 compared to the matched normal respiratory epithelium, highlighting the important role of these key drivers in tumorigenesis. Laura Soucek and his colleagues (Masso-Valles et al., 2020) suggested that MYC, MYCL and MYCN might be therapeutic targets for lung cancer and that elevated Myc levels were also associated with treatment resistance, there may be significant opportunities for the combination of Myc inhibitors with immunotherapies. It is well known that cancer occurrence is closely associated with the uncontrolled clonal proliferation of cells (Chung et al., 2019). As a well-known prooncogenic gene, MYC has been reported in mediating cell proliferation (Feist et al., 2018). However, its relationship between it and cell proliferation and tumor stemness in LUAD also needs to be further clarified. Our study showed a significant positive correlation between cell proliferation rate as well as tumor stemness and MYC score, and further highlights its non-negligible role in regulating LUAD cell proliferation and maintaining tumor stemness. Previous studies (Ireland et al., 2020; Patel et al., 2021) have revealed the key role of MYC in small cell lung cancer (SCLC) from a genomics perspective. Trudy G. Oliver et al. (Ireland et al., 2020) defined different SCLC molecular isoforms, based on the expression of ASCL1, NEUROD1, POU2F3, or YAP1. They used mouse and human models with time-series single-cell transcriptomic analysis to reveal the dynamic evolution of MYC-driven SCLC isoforms, finding that in neuroendocrine cells, MYC activated Notch to dedifferentiate tumor cells, promoting the temporal transition of SCLC from ASCL1 + to NEUROD1 + to YAP1 + state. The study by Hideo Watanabe and his colleagues (Patel et al., 2021)has also revealed the previously undescribed roles of the historically defined general oncogenes c-Myc and L-Myc for regulating lineage plasticity across molecular subtypes and histological subclasses. From the data currently available, MYC in SCLC seems to be studied more fully compared with LUAD. Therefore, it is still important to further investigate the potential role of MYC in LUAD from multi-omics data.

Overall, we used information from up to 1,600 samples of multiple LUAD cohorts to represent the important role of MYC signaling in LUAD from multiple dimensions of transcriptional profile expression, genomic instability, genetic alteration and regulation, immune microenvironment landscape, cell differentiation, and disease survival.This provides a valuable reference for deeply revealing the mechanism of cancer-promoting action of MYC in LUAD. However, like many other studies, the present study has some limitations. First, this study was a retrospective study and it was difficult to completely eliminate selective bias; second, although the important role of MYC in LUAD was described from multiple perspectives using multiple large study cohorts of LUAD and multiple bioinformatics approaches, further validation of the underlying experiments was lacking.

## Data Availability

The datasets presented in this study can be found in online repositories. The names of the repository/repositories and accession number(s) can be found in the article/Supplementary Material.
